# Exploring discontinuous intentions of social media users: a cognition-affect-conation perspective

**DOI:** 10.3389/fpsyg.2024.1305421

**Published:** 2024-02-02

**Authors:** Sara Qaisar, Anum Nawaz Kiani, Afsheen Jalil

**Affiliations:** ^1^International Islamic University, Islamabad, Pakistan; ^2^Rawalpindi Women University, Rawalpindi, Pakistan

**Keywords:** social media, depression, information overload, social overload, system feature overload, anxiety, cognition-affect-conation (C-A-C) framework

## Abstract

**Introduction:**

Drawing on the cognition-affect-conation (C-A-C) framework, this study investigates how perceived information and social and system feature overload induce depression and anxiety, which leads to affect discontinuous intentions of the social media users.

**Methods:**

The data collected from 570 social networking site users in China are analyzed through structural equation modeling (SEM).

**Results and Discussion:**

The findings show that perceived information overload, perceived social overload, and perceived system feature overload directly affect depression and anxiety among social networking site users, which directly leads to discontinuous intentions. This study fulfills the identified need for an in-depth investigation of discontinuous behavior in social networking sites. The findings provide social networking site providers with guidelines on how to actively manage social networking site user’s behavior to reduce the effects of negative emotions on social networking sites.

## 1 Introduction

Social networking sites (SNSs) are online service providers that aim to build social relationships among users who share common interests and activities (Luqman et al., 2017). SNSs provide a platform with good service quality, for example, by adding new features and functions that allow users to enhance their social lives and improve their social interactions with communication technology (Zhang et al., 2016). SNSs have enriched sources of information and the primary function of SNS is to share information among SNS users (Bright et al., 2015). Users invest a considerable amount of time in SNSs to expect improvement in their social relations and productivity with respect to communication technology (Choi and Lim, 2016). However, recent research has shown that users become depressed and their productivity is affected by the time spent using SNSs (Sagioglou and Greitemeyer, 2014). Many researchers have started investigating the negative side of SNSs usage and have reported that compulsive use of SNSs can result in negative emotional (i.e., depression and anxiety) and behavioral (i.e., discontinuous) consequences (Luqman et al., 2017; Dhir et al., 2018).

Many scholars have focused on determining the factors affecting the discontinuous intentions of SNS users. For example, Maier et al. (2015a) found that discontinuous behavior is a strategy to cope with stress and exhaustion. Discontinuous intentions refer to the behavioral intentions of users to reduce SNS usage, temporarily or permanently quit or switch to other SNSs (Ravindran et al., 2014; Maier et al., 2015b). Previous studies have highlighted the importance of stressful experiences and found that information, social, and system feature overload-induced psychological strains such as technostress, dissatisfaction, regret, exhaustion, and fatigue, which lead to user discontinuous intentions (Luqman et al., 2017; Cao and Sun, 2018; Nawaz et al., 2018; Cao et al., 2019; Rahmanyanti and Yasa, 2019).

Although limited studies have examined discontinuous behavior among social media users, several gaps remain for exploring discontinuous behavior in SNSs. First, extant studies have found that cognitive factors (i.e., perceived information and social and system feature overload) caused psychological strains (Choi and Lim, 2016; Lee et al., 2016; Zhang et al., 2016), thus the effect of these cognitive factors on user’s affective factors (i.e., depression and anxiety) remains unclear. Second, previous studies have found a direct relationship between compulsive SNSs usage (i.e., time spent on SNS and SNS checking frequency) with depression and anxiety (Dhir et al., 2018; Yoon et al., 2019; Chu et al., 2021) but the effect of user’s affective factors (i.e., depression and anxiety) on discontinuous intentions must be explored thoroughly. Third, studies have found the effect of information, social, and system feature overload on discontinuous intentions by drawing on stressor–strain–outcome (SSO) (Zhang et al., 2016; Cao and Sun, 2018; Qaisar et al., 2021) and stimuli–organism–response (SOR) framework (Luqman et al., 2017), thus these effects by drawing on cognition-affect-conation (C-A-C) framework remains unknown.

To fill the research gap, the current study explores the mechanism underlying the influence of cognitive and affective factors among SNS users on their intention to avoid using SNSs. We develop our research model based on the cognition-affect-conation (C-A-C) framework. Specifically, we assume that the perceived information, social, and system feature overload on SNSs induce negative emotions, i.e., depression and anxiety, thereby influencing SNS users to avoid using SNS and allowing them to adopt a strategy to exit from the situation.

This study exhibits three important contributions. First, we extend the applicability of the cognition-affect-conation (C-A-C) framework, which is an effective approach for examining customer loyalty in marketing, to the domain of the behavior of SNS users. Second, we extend previous studies that address discontinuous intentions from the perspective of overloads (e.g., information, social, and system features) and negative psychological factors (e.g., exhaustion, fatigue, dissatisfaction, and regret). Finally, this study strengthens the existing literature on emotions in information systems usage behavior research by explaining the vital role of negative emotions (i.e., depression and anxiety) among SNS users on their discontinuous intentions.

## 2 Literature review and hypothesis development

### 2.1 Cognition-affect-conation framework

The cognition-affect-conation model provides three basic stages of the mind, namely, cognitive (thinking or knowing), affective (emotions or feelings), and conative (doing or action). According to Huitt and Cain (2005), cognition refers to the perceptions, knowledge, and information that are acquired by the combination of direct experience. Affect refers to the emotions or feelings toward such perceptions and conation refers to the specific action or behavior of users.

Various studies have proposed and empirically confirmed theoretical models based on the C-A-C framework. For example, Hill et al. (1977) reveal that a user’s knowledge, beliefs, and opinions about an object generate favorable and unfavorable feelings toward that object, which demonstrate the user’s behavioral intentions or actions in the presence of a given object. The C-A-C framework is used in information system studies to find the user’s behavioral response, such as Davis (1989) described two cognitive beliefs (perceived usefulness and perceived ease of use) that affect their attitude (i.e., affection) and influence their intentions toward IT use (i.e., conation). Similarly, Huang et al. (2019) found that a person’s cognitive beliefs affect a user’s attitude, which directly enhances their intentions toward mobile health app usage. Dai et al. (2020) found that perceived information overload directly affects users’ emotions (frustration, fatigue, and dissatisfaction), thereby influencing their information avoidance intentions.

In the current study, we used the C-A-C framework as a theoretical framework to highlight the mechanism underlying the discontinuous intentions among SNS users. In the previous studies, the C-A-C model was used to analyze individual perception in the context of online services (Harris and Goode, 2004; Zhao et al., 2012; Lin, 2014). These studies verified the effectiveness of this model by identifying the sequential causal relationships among cognition, affect, and conation. Few studies have used other classic frameworks that are similar to C-A-C framework, such as stressor–strain–outcome (SSO) model (Lee et al., 2016; Zhang et al., 2016; Cao et al., 2018; Dhir et al., 2018; Nawaz et al., 2018; Fu et al., 2020; Qaisar et al., 2022), stimuli–organism–response (SOR) model (Luqman et al., 2017; Cao and Sun, 2018), and stress and coping theory to understand the influence of stress-related situations on the affective and behavioral outcomes of the users in the context of IT use. The purpose of using the C-A-C framework is that it aligns with the main objective of our research study, that is, to examine how the perceived information, social, and system feature overload of SNS users affects their affective (depression and anxiety) and conative (discontinuous intentions) responses.

### 2.2 Perceived information overload and depression

Social media improves individual social capital and subjective wellbeing, but the time spent on social media and the usage frequency of social media can have negative consequences once it exceeds from optimal level (Karr-Wisniewski and Lu, 2010). According to Moore (2000), overload is a key element in promoting negative consequences. Previous studies have described information overload occurs when users receive excessive information that affects their cognitive ability to process the information (Farhoomand and Drury, 2002; Eppler and Mengis, 2004; Carver and Turoff, 2007). That is, such overload transpires when the processing capabilities of users are exceeded by the information processing requirement. Previous research on social load theory implies that users experience cognitive limitation when their accessibility increases to a certain limit (Lee et al., 2016). Moreover, information overload refers to the situation when a user fails to process additional information due to its size and volume (Hoq, 2014). Zhang et al. (2016) described that information overload indicates the perceived amount of information that exceeds the processing capacity of the user. Similarly, the growing number of users on social media produces an excessive volume of information such as information about their friends and family, their personal lives, news, events, expertise, and rumors. These types of information attract intense attention from users, thereby inhibiting their cognitive ability with excessive information (Lee et al., 2016; Cao and Sun, 2018). Previous studies show that SNS users experience perceived information overload may result in negative behavioral consequences (Lee et al., 2016; Gao et al., 2018). Few studies confirmed that information overload exhibits a direct relationship with discontinuous usage intention (Dai et al., 2020; Zhang et al., 2020). Moreover, information overload also induced many psychological and emotional consequences, such as stress, exhaustion, regret, dissatisfaction, and SNS fatigue (Choi and Lim, 2016; Cao et al., 2018; Nawaz et al., 2018). Lee et al. (2016) found a direct relationship between information overload and SNS fatigue. Moreover, Dhir et al. (2018) reported that SNS fatigue is significantly related to depression. “Depression is an emotional state wherein pleasurable feelings are either diminished or disappeared.” Previous studies have found that depression has various psychological symptoms such as depressive moods and distress. Extant research studies found interference of depression in daily routine activities (i.e., sleep disorder, eating, loss of concentration, and fatigue) (Sapolsky, 2004; Bianchi et al., 2014). Few studies investigated depression in the context of social media and found that usage intensity and online communication caused the consequences of depression (Morrison and Gore, 2010; Cotten et al., 2013, 2014). Hussain et al. (2017), found that extreme smartphone social application usage results in high levels of depressive symptoms. Therefore, the current study expected that the perceived information overload of social media users directly influences their negative emotions (e.g., depression). Thus, the proposed hypothesis is as follows:

*H1(a)*. Perceived information overload has a positive relationship with depression.

### 2.3 Perceived information overload and anxiety

Anxiety is defined as a pervasive state of mind, which is concerned with difficult situations or threats (Bowers, 1986). Many scholars have found that anxious people believe that their ability to engage in certain tasks is detrimental (Madan et al., 2014). Previous studies have investigated the prevalence of anxiety among social media users. For example, Lepp et al. (2015) found that compulsive use of mobile social media applications exerts a direct impact on the anxious state of users. Social media requires deep involvement and concentration of users to participate in SNS activities (Can and Kaya, 2016). Excessive information on SNSs affects users’ psychological wellbeing (Chen and Li, 2017). Many studies have reported that information overload causes psychological distress, exhaustion, and emotional malfunctions (Hoq, 2014; Luqman et al., 2017; Swar et al., 2017). Psychology literature has suggested that anxious people are more likely to suffer from multiple disorders such as engaging in false judgment and unsystematic information processing (Kuss and Griffiths, 2011; Vermani et al., 2011; Stein and Sareen, 2015). Therefore, the current study expected that the perceived information overload of SNS users induces anxiety. Users with high perceived information overload are more likely to suffer from anxiety. Thus, the proposed hypothesis is as follows:

*H1(b)*. Perceived information overload has a positive relationship with anxiety.

### 2.4 Perceived social overload and depression

The number of social media relationships increases with the expansion of social media. Users become exhausted as they perceived to provide too much social support to their online friends. This phenomenon is known as social overload (Maier et al., 2015b). Users have to build and enhance their social contacts and interactions, which requires deep attention and more time to invest in maintaining social relationships, which leads to mental and psychological distress (McCarthy and Saegert, 1978). When social media requests are far more than the processing capability of users, the feeling of losing control over the social situation causes negative emotions. LaRose and Eastin (2004) defined that when the demands of maintaining and updating social media have detrimental effects on user’s lives. Previous studies have reported that social overload caused psychological distress due to a large number of social demands (Choi and Lim, 2016; Rahmanyanti and Yasa, 2019). According to Dunbar et al. (2018), there is a cognitive limit to the number of individuals with whom one can maintain stable relationships, which is approximately 150 users. Moreover, studies on Facebook reveal that a number of friends greater than Dunbar number decrease a user’s psychological wellbeing (Koc and Gulyagci, 2013; Bright et al., 2015; Li et al., 2015). Previous studies have revealed that social overload has a direct relationship with exhaustion, regret, and dissatisfaction (Maier et al., 2015b; Cao and Sun, 2018; Cao et al., 2019; Chu et al., 2021). Excessive social demands on SNSs may interrupt users’ attention and concentration from their daily work routine and evoke feelings of depression (Frison and Eggermont, 2015). Therefore, the current study expected that social overload exerts a direct impact on depression. Thus, we proposed the following hypothesis:

*H2(a)*: Perceived social overload has a positive relationship with depression.

### 2.5 Perceived social overload and anxiety

Previous research studies have found that perceived social overload usually involves deficient self-reaction to social demands and compulsive use of SNS, which contribute to negative affect such as stress (Lee et al., 2016; Cao et al., 2019; Fu et al., 2020; Qaisar et al., 2021). Moreover, social overload easily leads to low user satisfaction level and leads to social withdrawal (Lo et al., 2018). Maier et al. (2015b) found that users get tired of receiving too many virtual requests that limit their cognitive capacity to process and lead to influence negative emotions. Recently, a study found that compulsive use of SNS causes fatigue, which ultimately leads to anxiety (Dhir et al., 2018). Therefore, the current study expected that users with perceived social overload are more likely to be affected with anxiety.

*H2(b)*. Perceived social overload has a positive relationship with anxiety.

### 2.6 Perceived system feature overload and depression

System feature overload is defined as a situation when the technology is too complex to use by adding new features that increase its complexity (Karr-Wisniewski and Lu, 2010). Zhang et al. (2016) found that users frequently received system updates and modifications in SNSs. Too many features on SNS attract users to some extent but lead to feelings of strain such as fatigue and dissatisfaction. According to cognitive fit theory, unlimited and unnecessary features distract users’ attention and increase cognitive load, which leads to affect individual performance (Oviatt, 2006). The ultimate demands of SNS features require full attention to perceived usefulness, which, in turn, leads to psychological distress and exhaustion (Hsi and Potts, 2000; Thompson et al., 2005). The negative outcomes of system feature overload are due to the complexity of features, which requires more time to learn and decreases users’ perception of ease of use, which, in turn, leads to depression (Mick and Fournier, 1998). In this study, the researcher expected that perceived system feature overload exerts a direct impact on negative emotion (e.g., depression). Thus, the following hypothesis is proposed.

*H3(a)*. Perceived system feature overload has a positive relationship with depression.

### 2.7 Perceived system feature overload and anxiety

In the SNSs environment, new features and updates influence SNS users to a certain point but afterward decline. However, frequent changes occur in system features that are highly complex for users and could have negative consequences. When SNS users perceive the costs of learning and using the system features, they may get tired of SNS usage and feel fatigued (Zhang et al., 2016). Recent studies have linked fatigue and a state of anxiety (Dhir et al., 2018). Scholars have found that in users experiencing fatigue, cognitive capabilities decline, which inadequately regulates their mood and concentration. Particularly, researchers found that users experiencing fatigue and exhaustion are more likely to experience anxiety (Nawaz et al., 2018; Xie et al., 2018). Moreover, system feature overload limits the cognitive limit of users with too many features and functions, which increase negative emotions such as stress and anxiety (Lang, 2000; Li et al., 2014). Therefore, the current study expected that perceived system feature overloads influence negative emotions (e.g., anxiety). Thus, the following hypothesis is proposed.

*H3(b)*. Perceived system feature overload has a positive relationship with anxiety.

### 2.8 Discontinuous intentions and depression

Ravindran et al. (2014) defined three types of discontinuous intentions, namely, short breaks, control activities, and suspended behavior. Thus, in the SNS context, discontinuous intention is defined as an individual intention to decrease SNS use intensity, stop SNS, or switch to other alternative SNSs. Previous studies on SNSs have identified discontinuous intentions in stressful situations and users adopt this coping strategy to exit from the situation to restore their emotional stability (Maier et al., 2015a; Turel, 2015; Turel and Qahri-Saremi, 2016). Scholars have found that users who feel stressed will adopt behavioral coping strategies to exit from the situation (Turel and Serenko, 2012; Maier et al., 2015a; Tarafdar et al., 2019). Previous studies in psychology have demonstrated that information overload, system feature overload, and social overload induce psychological consequences that influence the discontinuous intentions of the users (Lee et al., 2016; Zhang et al., 2016; Cao and Sun, 2018; Dhir et al., 2018; Nawaz et al., 2018; Qaisar et al., 2022). Scholars argue that users with psychological, emotional, and behavioral consequences can experience depression (Sapolsky, 2004; Bashir and Bhat, 2017). Recent studies have found that intensive engagement with social media results in depression (Lin et al., 2016; Hussain et al., 2017). Moreover, depression can involve users in interpersonal and physiological difficulties (Scherr and Brunet, 2017). Moreover, many scholars have found that psychological and emotional consequences, such as exhaustion, regret, fatigue, and dissatisfaction, exhibit a positive effect on user’s discontinuous intentions (Luqman et al., 2017; Cao and Sun, 2018; Rahmanyanti and Yasa, 2019; Dai et al., 2020). Therefore, the current study expected that depression influences the discontinuous intentions of SNS users. Thus, the following hypothesis is proposed.

*H4*. Depression has a positive relationship with discontinuous intentions.

### 2.9 Discontinuous intentions and anxiety

**Figure 1:**
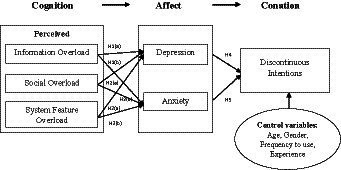
Research model.

Zhang et al. (2016) used the SSO framework to examine discontinuous intentions with perceived system features, information, and social overload as stressors that induce strain in terms of social network fatigue and dissatisfaction. Similarly, Cao and Sun (2018) described that information, communication, and social overload cause exhaustion and regret and ultimately affect the discontinuous intentions of users. Maier et al. (2015a) proposed that SNS stress creators and SNS exhaustion cause discontinuous intention, while switching stress creators and switching exhaustion reduce the intentions. Previous studies have investigated the antecedents and outcomes of anxiety such as psychological illness, emotional malfunctions, distress, and exhaustion (Kuss and Griffiths, 2011; Vermani et al., 2011; Baldwin et al., 2014). Users with high levels of exhaustion and fatigue are more likely to exit from the situation (Fu et al., 2020). Previous studies have revealed that SNS fatigue induced through compulsive use of SNSs exerts a direct impact on anxiety (Dhir et al., 2018). Lepp et al. (2014) found that compulsive mobile SNS users are more likely to suffer from anxious states as compared to non-compulsive users. Excessive social demand on SNSs reduces user capabilities, thereby triggering anxiety, and fatigue (Ravindran et al., 2014; Bright et al., 2015). Therefore, the current study expected that anxiety exerts a direct impact on users’ discontinuous intentions (Figure 1). Thus, the following hypothesis is proposed.

*H5*. Anxiety has a positive relationship with discontinuous intentions.

## 3 Methodology

### 3.1 Sample and data collection

The current research study analyses the mechanism of user’s intentions toward social media. Thus, the study context is confined to social media and the participants are social media users. Before collecting the data through the survey, the Back-translation method was used because the original questionnaire items were in English and translated into the Chinese language through Chinese translators for data collection; then, the questions were translated back into the English language for further analysis (Brislin, 1970). The instruments used in this study are well-established and underwent a translation process. Therefore, we conducted a pilot test and invited 50 volunteer students to fill out the questionnaire. The results showed that the measurement items were adequate for further implementation. Finally, the questionnaire was distributed online by sharing a link to the questionnaire among university students’ social groups (WeChat, Weibo, and QQ) as well as by sending invitations to students via university email. The targeted sample was based on SNS users because SNSs use in China has increased rapidly. Thus, SNS users are an adequate source of data for our study. All participants were assured that their data would remain confidential and that it was collected for research purposes only. A convenience sampling technique was used to collect data. The sample size for the unknown population was calculated through the criteria defined by Godden (2004); the recommended sample size was 384. A total of 596 survey responses were collected. After the elimination of outliers and incomplete responses, 570 responses were selected for further analysis.

### 3.2 Measures

Our study constructs were adapted from previous studies to test the proposed research model. All items were evaluated using a five-point Likert scale ranging from 1 = strongly agree to 5 = strongly disagree. Furthermore, sociodemographic data such as gender, age, frequency of use, and experience were also measured. The study measures are mentioned in Appendix A.

#### 3.2.1 Perceived information overload

The scale of perceived information overload was adapted from Zhang et al. (2016) and Karr-Wisniewski and Lu (2010). The four items of perceived information overload reflect the excessive amount of information available on SNSs. The Cronbach’s alpha (CA) value of the construct used in this study is 0.78.

#### 3.2.2 Perceived social overload

The scale of perceived social overload was adapted from Maier et al. (2015b). The four items of perceived social overload represent the need to provide social support to SNSs. The Cronbach’s alpha (CA) value of the construct used in this study is 0.78.

#### 3.2.3 Perceived system feature overload

The scale of perceived system feature overload was adapted from Karr-Wisniewski and Lu (2010). The four items of perceived system feature overload represent the addition of excessive features on SNSs. The Cronbach’s alpha (CA) value of the construct used in this study is 0.91.

#### 3.2.4 Discontinuous intentions

The discontinuous intention scale was adapted from Cao and Sun (2018) and van Offenbeek et al. (2013) to measure the behavioral patterns of SNS users. The Cronbach’s alpha value of the construct used in this study is 0.98.

#### 3.2.5 Depression

The scale of depression was adapted from Salokangas et al. (1995) and Dhir et al. (2018). The five items of depression represent the emotional state of SNS users. The Cronbach’s alpha (CA) value of the construct used in this study is 0.96.

#### 3.2.6 Anxiety

The scale of anxiety was adapted from La Greca and Lopez (1998) and Dhir et al. (2018). The five items of anxiety represent the user’s pervasive state of mind. The Cronbach’s alpha (CA) value of the construct used in this study is 0.93.

## 4 Data analysis and results

The data were analyzed through the following approaches. First, we used IBM SPSS 22 to perform exploratory factor analysis (EFA) to measure the reliability and validity (convergent and discriminant) of the constructs. Second, with the comprehensive technique of structure equation modeling (SEM), we used IBM AMOS 23 for confirmatory factor analysis (CFA) and determination of model fit indices of our proposed model. Finally, the research hypotheses were tested by examining the structural model.

Table 1 reveals the sociodemographic data of the respondents. Male respondents (52.6%) are more than female (47.4%) respondents. Among them, 47.7% of the respondents have more than 4 years of experience in using SNSs, and 41.6% uses SNSs many times a day.

**Table 1:** Demographics of respondents.

Category		Frequency	Percentage (%)
Gender	Male	300	52.6
	Female	270	47.4
Age	<18	12	2.1
	18–22	83	14.6
	23–27	276	48.4
	28–32	125	21.9
	33–37	46	8.1
	38–42	26	4.6
	>43	2	0.4
**Experience**			
	Less than 6 months	49	8.6
	6–12 months	59	10.4
	1–2 years	65	11.4
	3–4 years	127	22.3
	More than 4 years	270	47.4
**Frequency to use SNSs**			
	Many times a day	237	41.6
	Several times a day	194	34.0
	Once a day	82	14.4
	Less than once a day	57	10.0

### 4.1 Measurement model

#### 4.1.1 Reliability and validity

Validity pertains to how the constructs define the concept of the study, and reliability pertains to the component analysis produced four factors with an Eigen-value greater than 1, explaining 81.07% of the total variance. All factor loadings on the expected factor are within the range of 0.70 to 0.98 (Table 2), and the recommended values should exceed 0.7 to ensure construct validity (Hair et al., 1998). Second, to measure the reliability of the constructs, we used CA and composite reliability (CR) values. The values of CA and CR must exceed the threshold of 0.7 (Anderson and Gerbing, 1988). Table 2 indicates that all CA and CR values exceed 0.7, thereby ensuring measurement reliability. We also checked the average variance extracted (AVE) for each variable against its correlation with other variables to ensure convergent validity. In our data, all AVE values are greater than the minimum threshold of 0.50 as recommended by Fornell and Larcker (1981). Thus, these items satisfied the convergent validity requirements.

**Table 2:** Confirmatory factor analysis, AVE, and composite reliability.

Construct	Items	Factor loading	Cronbach’s alpha	AVE	CR
Information overload	IO1	0.750	0.78	0.56	0.83
	IO2	0.704			
	IO3	0.814			
	IO4	0.742			
Social overload	SO1	0.886	0.78	0.72	0.91
	SO2	0.888			
	SO3	0.783			
	SO4	0.840			
System feature overload	SYF1	0.898	0.91	0.78	0.94
	SYF2	0.900			
	SYF3	0.883			
	SYF4	0.867			
Depression	DP1	0.840	0.96	0.75	0.93
	DP2	0.775			
	DP3	0.856			
	DP4	0.930			
	DP5	0.936			
Anxiety	AX1	0.868	0.93	0.75	0.93
	AX2	0.829			
	AX3	0.843			
	AX4	0.889			
	AX5	0.909			
Discontinuous intentions	DI1	0.976	0.98	0.95	0.98
	DI2	0.983			
	DI3	0.973			

Third, we performed the discriminant validity of our constructs. Discriminant validity is the square root of the AVE and it should be greater than the correlation between it and all other constructs. Table 3 shows that all the values of the square root of AVEs are higher than outer correlations. This finding affirms discriminant validity. Furthermore, we used IBM AMOS 23 to conduct CFA to validate the measures. The CMIN/df = 1.76; NFI = 0.98; IFI = 0.97; TLI = 0.96; CFI = 0.98 and RMSEA = 0.03. The results indicated that the values are within the acceptable range as suggested by Hair et al. (1998). Therefore, the results show a valid model fit.

**Table 3:** Mean, standard deviation, and correlations.

	Mean	SD	IO	SO	SYF	DP	AX	DI
IO	3.79	1.033	**(0.75)**					
SO	4.14	0.749	0.36	**(0.85)**				
SYF	4.09	0.772	0.30	0.35	**(0.88)**			
DP	4.02	0.631	0.16	0.39	0.40	**(0.86)**		
AX	3.78	0.864	0.47	0.11	0.11	0.15	**(0.86)**	
DI	2.99	1.669	0.10	0.08	0.04	0.14	0.11	**(0.97)**

The diagonal elements (in bold) are the square root of variance shared between the AVE, whereas the off-diagonal elements are the correlations among constructs. IO, Information Overload; SO, Social Overload; SYF, System feature Overload; DP, Depression; AX, Anxiety; DI, Discontinuous Intentions.

#### 4.1.2 Common method bias

We performed Harman’s one-factor test to evaluate the extent of common method bias (Podsakoff et al., 2003) because all questions were answered by the same individual. In this test, the threat of common method bias is considered high if a single factor accounts for more than 50% of the total variance (Harman, 1976). The results reveal that none of the factors dominates the explanation of the variance, in which the most influential factor accounts for 27.0% of the variance. Moreover, other evidence of common method bias includes high correlations (*r* > 0.9) among variables (Pavlou and El Sawy, 2006). Table 3 shows no unusually high correlation in the sample. Thus, common method bias is not a serious concern in this study. Consistency of the construct is measured through reliability (Carmines and Zeller, 1979).

### 4.2 Structural model

Our study used IBM AMOS 23 to run the SEM. The basic purpose of SEM is to measure the fitness of the model. The results of the model fit indices show that the model has good fit [ $\chi^{2}\;\left(957.78\right)$ , df = 243, $\chi^{2}$ /df = 3.94, NFI = 0.94, IFI = 0.95, CFI = 0.95 and RMSEA = 0.07]. The proposed model is within the acceptable range defined by Anderson and Gerbing (1988), that is, $\chi^{2}/df$ < 5, NFI > 0.90, IFI > 0.90, CFI > 0.90 and RMSEA <1.0.

This study used SEM to test the proposed hypotheses. H1(a) states that the perceived information overload has a positive relationship with depression was supported ( β=0.09 , *p* < 0.001). H1(b) states that the perceived information overload has a positive relationship with anxiety was supported ( β=0.39 , *p* < 0.001). H2 (a) states that perceived social overload has a positive relationship with depression was supported ( β=0.33 , *p* < 0.001). Similarly, H2(b) states that perceived social overload has a positive relationship with anxiety was supported ( β=0.26 , *p* < 0.001). H3 (a) states that perceived system feature overload has a positive relationship with depression was supported ( β=0.32 , *p* < 0.001). H3(b) states that perceived system feature overload has a positive relationship with anxiety was supported ( β=0.12 , *p* < 0.001). Moreover, H4 states that depression has a positive relationship with discontinuous intention was supported ( β=0.38 , *p* < 0.001). H5 states that anxiety has a positive relationship with discontinuous intention was supported ( β=0.21 , *p* < 0.001). Furthermore, we used ANOVA to check the significant differences in control variables (gender, age, frequency to use, and experience). The control variables exhibit insignificant effects on discontinuous intentions. Therefore, we exclude the control variables for further analysis.

## 5 Discussion

### 5.1 Findings

On the basis of the cognitive–affect–conation (C-A-C) model, the current study proposed a model to examine the effects of overloads on the discontinuous intentions of users. Specifically, the study finds out the empirical linkage of perceived information, social and system feature overload with depression and anxiety that influence users’ discontinuous intentions. The study findings supported the proposed hypotheses. To the best of our knowledge, this study is the first to examine perceived information, and social and system feature overload in relation to depression, anxiety, and discontinuous intentions.

The current study offers important findings. First, the perception of SNS users toward information overload, social overload, and system feature overload exerts positive influences on their emotions such as depression and anxiety. The study results are in line with the previous study by Zhang et al. (2016), which revealed that perceived information overload, social overload, and system feature overload had a positive relationship with SNS fatigue and dissatisfaction. Similarly, Lee et al., 2016 that information overload exhibits a positive relationship with SNS fatigue. Fu et al. (2020) found that information overload, social overload, and system feature overload lead to an individual’s social media fatigue. Dhir et al. (2018) found that SNS fatigue exhibits a positive significant relationship with dissatisfaction and anxiety. Choi and Lim (2016) found that social overload leads to effect psychological wellbeing of the users. Moreover, Cao and Sun (2018) revealed that information overload, communication overload, and social overload lead to affect individual internal psychological processes, namely, exhaustion and regret.

Second, the study findings show that depression has a positive relationship with discontinuous intentions. The results are in line with the study of Swar et al. (2017), which found that an individual’s psychological ill-being (i.e., negative effect, depressive symptoms, trait anger, and trait anxiety) has a positive relationship with users’ discontinuous intentions. Similarly, Yoon et al. (2019) and Wang et al. (2019) found a positive significant relationship between SNS usage variables (i.e., time spent and frequency to use) and depression.

Finally, the current study found that anxiety has a positive relationship with discontinuous intention. The results are consistent with the study of Becker et al. (2012), which revealed that individuals who suffer from a decline in cognitive capabilities become anxious. Similarly, Dhir et al. (2018) and Hussain et al. (2017) found that compulsive use of SNS causes SNS fatigue, which leads to detrimental consequences (i.e., anxiety). Users with high levels of exhaustion and fatigue are more likely to exit from the situation (Fu et al., 2020).

### 5.2 Theoretical implications

The study findings have several important theoretical implications. First, the current study extends the research on the negative effects of SNSs use because limited research was conducted on it which has to be explored thoroughly. Our work expands the previous studies that examined the effect of overloads on discontinuous intention and offers a more comprehensive understanding of the dark side of SNSs. Second, previous research studies have described the C-A-C model to understand the sequential casual relationships among cognition, affect, and conation, which has been confirmed by empirical studies of e-commerce, marketing, and information systems. To date, this model is merely utilized in the context of discontinuance among SNSs users. The current study extends the application of the C-A-C framework in discontinuance of SNSs usage. As expected, their cognition (i.e., perceived information and social and system feature overload) exhibits a positive significant effect on their affection (i.e., depression and anxiety), thus affecting their conation (i.e., discontinuous intention). Third, previous studies have examined the effect of information overload, social overload, and system feature overload on user’s psychological wellbeing and find out psychological factors, such as exhaustion, regret, fatigue, and dissatisfaction (Maier et al., 2015a; Luqman et al., 2017; Cao and Sun, 2018; Nawaz et al., 2018). The current study contributes to the existing literature and finds out the effect of information overload, social overload, and system feature overload on depression and anxiety which the previous studies have not examined. Finally, the study examines the negative emotional reactions of individuals, particularly depression and anxiety, thereby substantially contributing to the research on discontinuous use. The study complements the previous studies and extends the literature on discontinuous intention. Our results reveal that depression and anxiety are critical antecedents to the users’ discontinuous intentions.

The current study has several important practical implications. First, perceived information overload, social overload, and system feature overload are the predictors of depression and anxiety. The SNS providers should filter the information available on SNSs such as content personalization and also limit friends’ social circles, and social activities to reduce the effects induce by social overload. Moreover, SNS providers should provide detailed guides about new features to the SNS users to avoid the negative consequences induced through the addition of new features and also remove unnecessary features. Second, our findings demonstrate that depression and anxiety are the key factors of user’s discontinuous intention. Therefore, SNS providers and social marketers on SNS should provide a new mechanism that could effectively prevent users from producing negative emotions. Moreover, SNS users must control their usage and remove unnecessary friends to reduce the emotional consequences. Finally, SNS providers must provide training to SNS users about how to efficiently use SNS and how to effectively extract useful information to get benefits to reduce the negative behavioral consequences.

### 5.3 Limitation and future work

This study poses several limitations. First, data were collected from university students, which was the best fit for our research study because it is a represented sample of SNSs in China. It is an empirical question as to whether the findings can be generalized to other countries and cultures. Various cultural factors, values, and beliefs have an impact on individual psychological wellbeing (Wissing and Temane, 2008; Grossi et al., 2012). Future research should examine this model in different settings (i.e., organizations), with other samples (i.e., working professionals and users with low education levels), and in different countries to ascertain interesting results. Second, this study was single-center, and the convenience sampling method was used to limit the diversity of results, so the results may not represent the total number of SNSs users to find their discontinuance intentions. Future studies should use some other sampling techniques such as snowball or cluster sampling technique to find out more diverse results. Third, the current study analyzed three cognitive factors (i.e., perceived information overload, social overload, and system feature overload) and two affective factors (i.e., depression and anxiety) in the model. Several other cognitive (i.e., perceived usefulness, perceived information quality, and perceived technology overload) and affective (i.e., envy and sleep disorder) factors may affect discontinuous intentions. Future studies should address the other factors in our model that lead to discontinuous intentions. Finally, the current study did not focus on any specific SNSs because most of the SNSs have similar features and functions, the context of the study is general. Future studies should focus on a comparative study of popular SNSs to find out different results.

## Data availability statement

The raw data supporting the conclusions of this article will be made available by the authors, without undue reservation.

## Ethics statement

Ethical review and approval was not required for the study on human participants in accordance with the local legislation and institutional requirements. Written informed consent from the participants or participants legal guardian/next of kin was not required to participate in this study in accordance with the national legislation and the institutional requirements.

## Author contributions

SQ: Writing – original draft. AN: Data curation, Formal analysis, Methodology, Writing – review & editing. AJ: Methodology, Resources, Validation, Writing – review & editing.

## Appendix A

Measures and operationalisation of constructs.

**Table:**

Construct	Item wordings	Source
Information overload	(IO1) I am often distracted by the excessive amount of information in SNSs	Adapted from Zhang et al. (2016) and Karr-Wisniewski and Lu (2010)
	(IO2) I am overwhelmed by the amount of information that I process on a daily basis from SNSs	
	(IO3) I feel some problems with too much information in SNSs to synthesize instead of not having enough information	
	(IO4) There is too much information about my friends on SNSs so I find it a burden to handle	
Social overload	(SO1) I take too much care of my friends’ well-being on SNSs	Adapted from Maier et al. (2015b)
	(SO2) I deal too much with my friend’s problems on SNSs	
	(SO3) I am too often caring for my friends on SNSs	
	(SO4) I pay too much attention to my friends’ posts on SNSs	
System feature overload	(SYF1) I am often distracted by features that are included in SNSs but are not related to my main purpose in using SNSs	Adapted from Karr-Wisniewski and Lu (2010)
	(SYF2) I find that most features of SNSs contain too many poor subfeatures instead of too few very good subfeatures	
	(SYF3) SNSs tends to try to be too helpful by adding features, which makes social performance even harder	
	(SYF4) The features of SNSs I use are often more complex than the tasks I have to complete using these features	
Depression	(DP1) I have felt lonely	Adapted from Salokangas et al. (1995) and Dhir et al. (2018)
	(DP2) I did not enjoy my life	
	(DP3) I have felt myself unworthy	
	(DP4) I have felt all the joy had disappeared from my life	
	(DP5) I have felt my sadness was not relieved even with help of family/friends	
Anxiety	(AX1) I worry about what others say about me	Adapted from La Greca and Lopez (1998) and Dhir et al. (2018)
	(AX2) I worry that others do not like me	
	(AX3) I am afraid that others make fun of me	
	(AX4) I worry about what others think of me	
	(AX5) I feel that others make fun of me	
Discontinuous Intentions	(DI1) I intend to stop using SNSs.	Adapted from Cao et al. (2018) and van Offenbeek et al. (2013)
	(DI2) I predict that I would reduce my SNSs use.	
	(DI3) I plan to stop or reduce my SNSs use.	
